# Attitudes and Perception of Healthcare Workers Concerning Influenza Vaccination during the 2019/2020 Season: A Survey of Sicilian University Hospitals

**DOI:** 10.3390/vaccines8040686

**Published:** 2020-11-16

**Authors:** Claudio Costantino, Caterina Ledda, Raffaele Squeri, Vincenzo Restivo, Alessandra Casuccio, Venerando Rapisarda, Giorgio Graziano, Davide Alba, Livia Cimino, Arianna Conforto, Gaetano Bruno Costa, Smeralda D’Amato, Francesco Mazzitelli, Francesco Vitale, Cristina Genovese

**Affiliations:** 1 Department of Health Promotion Sciences, Maternal and Infant Care, Internal Medicine and Medical Specialties (PROMISE) “G. D’Alessandro”, University of Palermo, 90127 Palermo, Italy; claudio.costantino01@unipa.it (C.C.); vincenzo.restivo@unipa.it (V.R.); alessandra.casuccio@unipa.it (A.C.); giorgio.graziano@gmail.com (G.G.); davide.alba@unipa.it (D.A.); livia.cimino@unipa.it (L.C.); arianna.conforto@unipa.it (A.C.); francesco.vitale@unipa.it (F.V.); 2 Occupational Medicine, Department of Clinical and Experimental Medicine, University of Catania, 95100 Catania, Italy; cledda@unict.it (C.L.); vrapisarda@unict.it (V.R.); 3 Department of Biomedical Sciences and Morphological and Functional Images (BIOMORF), University of Messina, 98124 Messina, Italy; squeri@unime.it (R.S.); gbcosta@unime.it (G.B.C.); damato.esmeralda@libero.it (S.D.); francesco88xp@libero.it (F.M.)

**Keywords:** influenza vaccine, flu vaccination, healthcare workers, refusal, adherence, attitudes

## Abstract

Influenza is an infectious disease with a high impact on the population in terms of morbidity and mortality, but despite International and European guidelines, vaccination coverage rates among healthcare workers (HCWs) remain very low. The aim of the present study was to evaluate influenza vaccination adherence in the three Sicilian University Hospitals of Catania, Messina, and Palermo and to understand the attitudes and perceptions of vaccinated healthcare workers and the main reasons for vaccination refusal. A cross-sectional survey through a self-administered questionnaire was conducted during the 2019/2020 influenza season. Overall, 2356 vaccinated healthcare workers answered the questionnaire. The main reason reported for influenza vaccination adherence during the 2019/2020 season was to protect patients. Higher self-perceived risk of contracting influenza and a positive attitude to recommending vaccination to patients were significantly associated with influenza vaccination adherence during the last five seasons via multivariable analysis. Fear of an adverse reaction was the main reason for influenza vaccine refusal. In accordance with these findings, Public Health institutions should develop and tailor formative and informative campaigns to reduce principal barriers to the immunization process and promote influenza vaccination adherence among HCWs.

## 1. Introduction

Influenza (flu) is an infectious disease with a high impact on the population in terms of morbidity and mortality, particularly among risk categories such as the elderly, people with comorbidities, pregnant women, and young children [1,2,3]. However, in Italy, the flu vaccination coverage rates in high-risk groups were considerably low during the 2018/2019 season [4]. In this context, healthcare workers (HCWs) play a fundamental role as behavior models and a reliable source of advice for patients, especially in the healthcare context, as they can become spreaders of influenza and other vaccine-preventable disease (VPD) infections among patients [5,6,7,8]. In addition, influenza vaccination of HCWs is recommended during seasonal epidemics of influenza in order to limit absenteeism among personnel and subsequent disruption of healthcare services [9].

Despite International and European guidelines, which strongly recommend seasonal flu vaccination and vaccination against other VPDs among healthcare workers, coverage rates remain very low [10,11,12,13]. In particular, the flu vaccination coverage rates in 12 European countries during the last seasons ranged from 15.6% to 63.8%, with a median value in 2016/2017 of 30.2% [14]. The highest vaccination coverage rates were reported in Belgium, Wales, and England, where 70.3% of HCWs received the seasonal influenza vaccine during the 2018/2019 season [15,16,17,18]. Of interest, flu vaccination adherence among Sicilian HCWs ranged from 24.3% to 37.2% during the last influenza season [19].

Several studies have analyzed and discussed the main reasons associated with influenza vaccination refusal among HCWs [15,17]. During the last decade, general higher confidence and awareness among younger healthcare employees, such as medical residents/students and trainees, has been observed, resulting in better vaccination coverage rates [9,10]. Moreover, correct knowledge, attitudes, and practices regarding flu infection and the vaccination of younger HCWs supported an increase in vaccination adherence among older workers as well [17,18]. 

The main aims of the present study were to evaluate vaccination adherence in the three Sicilian University Hospitals of Catania (UHC), Messina (UHM), and Palermo (UHP), and to understand the attitudes and perceptions of vaccinated HCWs and the main reasons for vaccination refusal.

## 2. Materials and Methods 

### 2.1. Data Collection 

A cross-sectional survey through a self-administered questionnaire was conducted during the 2019/2020 influenza season, between November 2019 and January 2020, among vaccinated HCWs working in UHC, UHM, and UHP, accounting for 2670, 2340, and 2720 employees (including medical residents), respectively. Moreover, the dissent forms of unvaccinated HCWs at the three Sicilian University Hospitals (UHs) were analyzed, and the flu vaccination coverage rates observed during the last four influenza seasons (from 2016/2017 to 2019/2020) were also analyzed. 

Palermo, Catania, and Messina are the three major Sicilian cities, accounting for 663,770, 311,777, and 231,708 inhabitants, respectively, and are the only cities with a University Hospital in the Sicilian Region. The main characteristics of the flu vaccination campaign of the 2019/2020 season in the Sicilian administrative region were based on decree n. 1829 of 20 September 2019 [20]. In detail, different to previous influenza seasons, all HCWs that refused an influenza vaccination were required to wear a personal protective mask at all times during working hours to protect fragile patients [20]. 

Moreover, similarly to previous influenza seasons (2017/2018 and 2018/2019), all HCWs that did not receive a flu vaccination were required to fill in a dissent form, specifying the main reason for vaccine refusal. 

The questionnaire administered to HCWs vaccinated against influenza during the 2019/2020 flu season at the Sicilian UHs was divided into the following sections: 

(a) Socio-demographic data: age, gender; 

(b) Working activity data: type of healthcare professional and hospital units (divided into “at risk” and “not at risk” according to the medical conditions of hospitalized patients, which affect the probability of contracting an infection); 

(c) Daily washing of hands (≤3 times a day, 4–6 times a day, or ≥7 times a day); 

(d) Vaccination adherence during the last five seasons (divided into never vaccinated, vaccinated 1 or 2 times, and regularly vaccinated (3–5 times)); 

(e) Self-perceived risk of contracting influenza in comparison with the general population (equal, higher, or lower); 

(f) Considering themselves as a high-risk group to infect patients with influenza virus (yes; yes, partially; or no); 

(g) Main reason for influenza vaccination adherence (to protect themselves, to protect patients, or to avoid compulsory mask wearing); 

(h) Attitude to recommending influenza vaccination to patients (yes or no). 

In detail, a hospital unit was considered “at risk” if the majority of the patients admitted at the same ward were affected by at least one severe chronic medical condition (such as cardiology, neurology, pulmonology, or intensive care units).

At the same time, the responses to the online dissent form filled in by all healthcare professionals working at the UHs of Catania, Messina, and Palermo who refused an influenza vaccination during the 2019/2020 season were analyzed [21].

Finally, the influenza vaccination coverage rates during the last four influenza seasons (from 2016/2017 to 2019/2020) were calculated, excluding students and trainees from the computation. This choice was due to the different policy of the Catania UH, in comparison to that recommended at the Messina and Palermo UHs, which does not encourage flu vaccination of students/trainees. 

The methods used to ensure the confidentiality of data were explained, and written informed consent was obtained from all participants, according to the Italian privacy law. Ethical approval was obtained from the Ethics Committee of the University Hospital of Palermo (Palermo, Italy) in October 2019 (n.09/2019). 

### 2.2. Statistical Analysis 

The absolute and relative frequencies were calculated for categorical variables, while continuous variables were summarized as median (interquartile range). Differences between groups for categorical variables were analyzed using the Chi-square test (Mantel–Haenszel). The significance level chosen for all analyses was set at *p* < 0.05 (two-tailed). 

The Crude Odds ratio (OR) and Adj OR of variables associated with at least one flu vaccination carried out during the last five influenza seasons were calculated. In this analysis, students and trainees (belonging to UHM and UHP) were excluded because they were more likely to be vaccinated for the first time during the 2019/2020 influenza season. In the multivariate analysis, all variables that in the univariate analysis had a *p*-value of >0.20 were excluded. 

All the information collected through the questionnaire was entered into an electronic database created using Excel 16.0 software (Microsoft Corporation, Redmond, WA, USA). Data analysis was performed using STATA14^®^ software (StataCorp, Lakeway, TX, USA). 

## 3. Results

Overall, 2356 vaccinated healthcare professionals answered the questionnaire at the UHC, UHM, and UHP (response rates among all vaccinated HCWs: 67.5%, 68.3%, and 74.1%, respectively), as reported in Table 1. The mean age of respondents was 33.5 years (SD: ±13.1) among the HCWs at UHP, 29.1 years (SD: ±10.8) among the HCWs at UHM, and 39.3 years (SD: ±12.6) among the HCWs at UHC (*p* < 0.001). There were no significant differences in gender (an overall prevalence of female HCWs was reported in all three Sicilian UHs) or work setting (working in at-risk or not-at-risk hospital units). 

The most represented category was student/trainees at UHP and UHM, accounting respectively for 44.5% and 59.3% of the HCWs enrolled. At UHC, those enrolled were mostly medical doctors (68.8%), while as specified in the methods section, healthcare students and trainees were not surveyed. This could be associated with the significantly higher mean age of HCWs enrolled at the Catania UH, in comparison with UHM and UHP.

In Table 2, the preventive attitudes of HCWs working in the three Sicilian University hospitals and vaccinated against influenza are reported. A large majority of HCWs washed their hands more than seven times a day: 681 (58.4%), 230 (52.4%), and 577 (77.3%), respectively, for UHP, UHM, and UHC (*p* < 0.001). 

Concerning previous influenza vaccination adherence, more than 50% of HCWs at UHP and UHM (56.9% at Palermo and 69.1% at Messina) declared having been vaccinated one or two times (rarely) or at least three times (regularly) during the last five influenza seasons. The HCWs at UHC declared having never been vaccinated during the previous five influenza seasons at a significantly higher rate (*n* = 569; 76.3%) (*p* < 0.001). 

Self-perceived risk of contracting influenza in comparison with the general population was considered higher more frequently by HCWs working at UHP and UHM (66.4% and 69.6%, respectively) than by HCWs at UHC (31.9%) (*p* < 0.001). 

Almost half of the HCWs at UHP (78.6%) and Messina (73.4%) considered themselves a high-risk group to infect patients with influenza virus, in comparison to 32.7% at UHC (*p* < 0.001). The main reason reported by HCWs for influenza vaccination adherence during the 2019/2020 season was to protect patients (66.5% at UHP, 65.9% at UHM, and 50.2% at UHC). 

Significantly higher percentages of HCWs working at UHP (85.3%) and UHM (87.3%), in comparison with HCWs at UHC (37.2%), usually recommend influenza vaccination to their own patients (*p* < 0.001).

In Table 3 are reported the results of univariate/multivariate analysis between regular vaccination against influenza among HCWs (at least one time during the last five influenza seasons) and the other variables studied. In the multivariate analysis, significant associations were found between at least one influenza vaccination during the last five influenza seasons and higher self-perceived risk of contracting influenza in comparison with the general population (Adj OR = 2.65; Confidence Interval 95%: 1.89–3.65) and attitude to recommending influenza vaccination to patients (Adj OR = 3.45; Confidence Interval 95%: 1.78–5.63).

**Figure 1:**
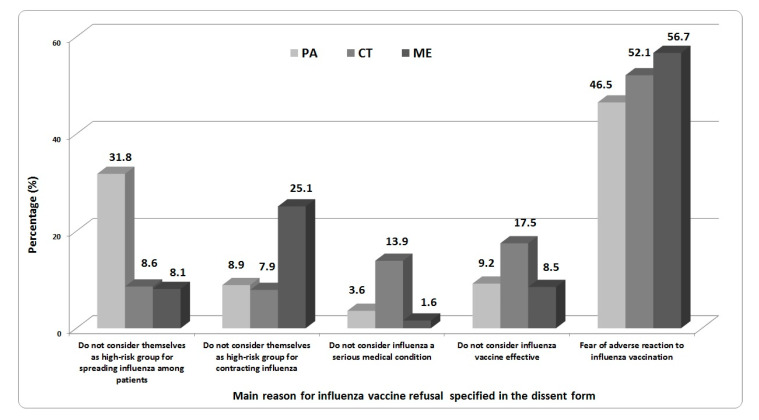
The main reasons for influenza vaccine refusal specified in the dissent form by HCWs (excluding students/trainees) at the Catania, Messina, and Palermo UHs. (Students and trainees did not fill in the dissent form, in accordance with the Sicilian Regional Decree).

Overall, 2567 dissent form were analyzed (695 from the UHP, 1253 from UHC and 619 from UHM). As reported in Figure 1, fear of an adverse reaction was the main reason for influenza vaccine refusal specified by HCWs at all three Sicilian UHs in the dissent form (46.5% at UHP; 52.1% at UHC; 56.7% at UHM). Moreover, at UHP, 31.8% of HCWs do not consider themselves a high-risk group for spreading influenza among patients, while 25.1% of HCWs at UHM do not consider themselves a high-risk group for contracting influenza. More than 30% of HCWs at UHC consider influenza to not be a serious medical condition or consider the influenza vaccine to be ineffective.

**Figure 2:**
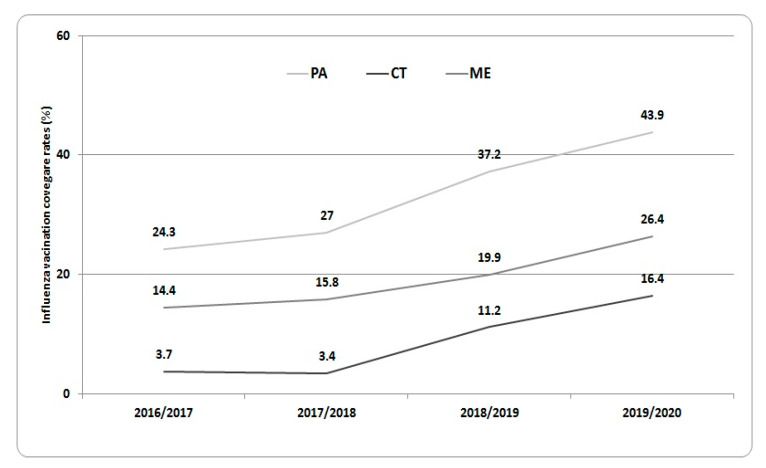
Influenza vaccination coverage rates against seasonal influenza observed during the last four influenza seasons (from 2016/2017 to 2019/2020) at the UHs of Catania, Messina, and Palermo (students and trainees were removed from analysis).

Finally, in Figure 2, the influenza vaccination coverage rates observed among HCWs of the three Sicilian UHs during the last four influenza seasons (2016/2017–2019/2020) are shown. Influenza vaccination adherence significantly increased from 24.3% in 2016/2017 to 51.8% in the 2019/2020 season at UHP, from 14.4% in 2016/2017 to 26.4% in the 2019/2020 season at UHM, and from 3.7% in 2016/2017 to 16.4% in the 2019/2020 season at UHC. 

## 4. Discussion

Influenza vaccination adherence among healthcare workers has shown a slow but constant increase during previous vaccination campaigns in the Italian context [22,23]. 

Nevertheless, coverage rates remain very low in comparison to those recommended by National and International Health Authorities but are in line with data described by other European investigations [24,25,26]. 

The different vaccination coverage rates observed among three Sicilian UHs could be attributed to the different dynamics of invitation to and administration of influenza vaccinations [27]. 

In particular, the Messina and Palermo UHs have a dedicated vaccination unit for healthcare workers, while at the University Hospital of Catania, flu vaccinations are administered at the occupational medicine unit [9,19]. 

Moreover, at the Palermo UH, which showed the highest influenza vaccination coverage rates among HCWs of the entire Sicilian Region, several initiatives have been promoted during previous influenza seasons in order to increase vaccination adherence [9]. The distribution of pins (with a logo stating “I’m vaccinated”) to vaccinated HCWs, dedicated “flu vaccination days” to promote influenza vaccination through open conferences, posters and flyers promoting influenza vaccination uptake (with the slogan “Protect yourself to protect your patients”), dedicated pages on the institutional website and social networks of the Palermo UH, and “on-site vaccination” are only some of the strategies adopted at the UH of Palermo [9].

Regarding the different attitudes observed at the three Sicilian UHs, lower vaccination adherence during the previous five influenza seasons, lower self-perceived risk of contracting influenza in comparison with the general population, and lower consideration of themselves as a high-risk group to infect patients could be attributed to the higher prevalence of older HCWs at the UH of Catania, where medical students and trainees were not enrolled. 

Higher vaccination coverage rates and adherence in the youngest age classes are important to limiting the spread of influenza viruses among patients, but, at the same time, the low vaccination coverage reported among older HCWs could represent a public health issue, because they are a group at increased risk of developing severe and complicated influenza infections [7,28,29].

As a matter of fact, the significant association found between more frequent vaccination during previous seasons among HCWs and higher self-perceived risk of contracting influenza in comparison with the general population confirms what has been reported previously in other studies [7,17,18]. 

Furthermore, the attitude to recommending influenza vaccination to patients among HCWs more frequently vaccinated against influenza in the past could support a key role of seasonal influenza vaccination among HCWs in increasing vaccination adherence among the general population [23,30].

Several studies performed in recent years have tried to evaluate the main factors associated with low adherence to influenza vaccination among HCWs in order to design more active policies to improve immunization rates in this high-risk category [31,32,33,34,35].

In general, as reported also in the present survey, a fear of adverse events is the main reason for influenza vaccination refusal among HCWs. 

The authors of a review of 25 studies on attitudes toward and predictors of flu vaccination among HCWs recognized two main reasons for the lack of vaccine acceptance by HCWs: first, an extensive series of misconceptions or absence of information about influenza infection, and secondly, an absence of appropriate access to vaccination [36]. 

On the other hand, a study carried out in Italy established that self-protection and protection of family members and further persons close to HCWs are the main drivers motivating HCWs toward influenza vaccination adherence [35]. 

From this perspective, all the initiatives and strategies adopted in healthcare contexts to improve influenza vaccination knowledge, attitudes, and perceptions could contribute to an increase in adherence [37,38,39,40]. Palermo UH adopted several strategies in previous seasons that contributed to a reduction in vaccine hesitancy and considerably increased vaccination adherence among HCWs [9]. 

Numerous reviews of observational studies and trials that have assessed the efficiency of one or more interventions to improve adherence to influenza vaccination among HCWs have illustrated that campaigns with multiple strategies are associated with a higher probability of vaccine acceptance [36,40,41]. 

Although information on the diffusion of “vaccine hesitancy” among HCWs is not available, the European Centre for Disease Prevention and Control (ECDC) investigated this phenomenon and observed that the major elements among European HWCs are, different from those among the general population, apprehensions about vaccine safety, especially the flu vaccine, and suspicion of the pharmaceutical industry, governments, health authorities, and research [42,43]. 

Furthermore, a possible strategy to be considered to increase HCWs’ influenza vaccination adherence is mandatory vaccination, which was introduced in the USA with excellent results: as described by a CDC Internet panel survey, HCW influenza vaccination coverage during the 2018–2019 influenza season was 81.1%, with the highest coverage (97.7%) among HCWs with workplace vaccination requirements and the lowest (42.1%) among those working in settings where vaccination was not required, promoted, or offered on-site [44]. 

Actually, mandatory vaccinations against vaccine-preventable diseases for HCWs, recently suggested by many authors for European and Italian settings, have been introduced in the Regional decrees of three Italian Regions (Emilia Romagna, Marche, and Apulia) [44,45]. 

Finally, during the next influenza season, an overlap of influenza viruses with SARS-Cov-2 and the recent evidence of a possible cross-protective effect of flu vaccination on limiting COVID-19 morbidity and lethality could further increase vaccination adherence among HCWs [46,47]. 

Some limitations of this study need to be highlighted. Readers should consider selection bias due to the responses to the survey being only from HCWs who accepted influenza vaccination. At the same time, HCWs who refused the influenza vaccination were obliged to fill in the mandatory dissent form specifying only the main reason for flu vaccine refusal, making a comparison of the two samples of HCWs impossible. 

Moreover, a possible lack of representativeness of the sample, especially at the UH of Catania (where students/trainees were not vaccinated), could be taken into account. For this reason, students and trainees were excluded from multivariable analysis, dissent form analysis, and flu coverage rate analysis. On the other hand, the high number of participants in the survey from all three UHs involved represents a strength of the present study.

## 5. Conclusions

In conclusion, data obtained from the present study contributed to an analysis of the main factors associated with adherence to or refusal of the seasonal influenza vaccination among HCWs at the three Sicilian UHs. Higher self-perceived risk of contracting influenza relative to the general population and a positive attitude to recommending influenza vaccination to patients were found to be associated with a better adherence to flu vaccination over previous seasons. 

On the other hand, fear of an adverse reaction and not considering themselves a high-risk group for spreading influenza to patients were the main reasons reported for vaccine refusal. 

In accordance with these findings, future promotion and formative campaigns among HCWs should be customized in order to overcome the emerging barriers to the flu immunization process among HCWs. 

Certainly, educational and promotion programs, in addition to specific occupational counselling, should aim to eradicate some existing misconceptions among HCWs (such as those of adverse events from vaccination) which may limit their adherence to flu vaccinations. If, after the implementation of these interventions, flu vaccination adherence among HCWs remains lower than recommended, legislators should consider introducing a vaccination mandate for all HCWs.

## Figures and Tables

**Table 1:** Univariate analysis of the socio-demographics and working characteristics of healthcare workers (HCWs) vaccinated against influenza at the three Sicilian University Hospitals (UHs) (*n* = 2356).

Variables	Palermo UH (*n* = 1169) *	Messina UH (*n* = 440) *	Catania UH (*n* = 747) *	*p*-Value
Mean age ± Standard deviation	33.5 ± 13.1	29.1 ± 10.8	39.3 ± 12.6	<0.001
Median age (Interquartile range)	28 (25–40)	25 (22–32)	33 (29–50)	<0.001
Gender, *n* (%)				
- male	477 (40.9)	176 (40.0)	344 (46.1)	>0.05
- female	690 (59.1)	264 (60.0)	403 (53.9)	
HCW type, *n* (%)				
- medical doctor	429 (38.1)	134 (30.5)	514 (68.8)	<0.001
- nurse/healthcare assistant	195 (17.3)	45 (10.2)	233 (31.2)	
- student/trainee	501 (44.5)	261 (59.3)	0 (0.0)	
Hospital unit, *n* (%)				
- at risk	448 (71.1)	75 (74.3)	528 (70.7)	<0.76
- not at risk	182 (28.9)	26 (25.7)	219 (29.3)	

* For any variable, observations may not correspond due to missing data.

**Table 2:** Univariate analysis of the preventive attitudes of HCWs vaccinated against influenza at the three Sicilian University Hospitals (*n* = 2356).

Variables	Palermo UH (*n* = 1168) *	Messina UH (*n* = 440) *	Catania UH (*n* = 747) *	*p*-Value
Daily washing of hands, *n* (%)				
- ≤3 times a day	25 (2.1)	13 (2.9)	28 (3.7)	<0.001
- 4–6 times a day	462 (39.5)	196 (44.7)	142 (19.0)	
- ≥7 times a day	681 (58.4)	230 (52.4)	577 (77.3)	
Vaccination adherence during last five seasons, *n* (%)				
- Never vaccinated	500 (43.1)	136 (30.9)	569 (76.3)	<0.001
- Vaccinated 1 or 2 times	337 (29.1)	213 (48.4)	81 (10.8)	
- Regularly vaccinated (3–5 times)	323 (27.8)	91 (20.7)	97 (12.9)	
Self-perceived risk of contracting influenza in comparison with general population, *n* (%)				
- Equal	333 (28.8)	108 (24.5)	502 (67.3)	<0.001
- Higher	768 (66.4)	306 (69.6)	239 (31.9)	
- Lower	56 (4.8)	26 (5.9)	6 (0.8)	
Considering themselves as a high-risk group to infect patients with influenza virus, *n* (%)				
- Yes	352 (30.5)	123 (27.9)	94 (12.6)	<0.001
- Yes, partially	556 (48.1)	200 (45.5)	150 (20.1)	
- No	247 (21.4)	117 (26.6)	503 (67.3)	
Main reason for influenza vaccination adherence, *n* (%)				
- To protect themselves	375 (32.2)	142 (32.3)	151 (49.8)	<0.001
- To protect patients	775 (66.5)	290 (65.9)	152 (50.2)	
- To avoid compulsory mask wearing	15 (1.3)	8 (1.8)	0 (0.0)	
Attitude to recommending influenza vaccination to patients, *n* (%)				
- Yes	988 (85.3)	384 (87.3)	278 (37.2)	<0.001
- No	171 (14.7)	56 (12.7)	469 (62.8)	

* For any variable, observations may not correspond due to missing data.

**Table 3:** Crude Odds ratio (OR) and Adj OR and related Confidence interval (CI 95%) variables associated with at least one influenza vaccination during last five influenza seasons at the three Sicilian University Hospitals (students and trainees were removed from the multivariable analysis).

Variables	Crude OR	CI 95%	*p*-Value	Adj OR	CI 95%	*p*-Value
Gender						
- Male	ref		0.09	ref		0.12
- Female	0.70	(0.52–1.05)		0.86	(0.75–1.22)	
Age in years	1.45	(0.92–2.60)	0.08	1.59	(0.86–2.03)	0.15
HCWs type						
- nurse/healthcare assistant	ref		0.06	ref		0.18
- medical doctor	0.84	(0.6–1.01)		0.77	(0.52–1.12)	
Self-perceived risk of contracting influenza in comparison with general population						
- Equal or Lower	ref		<0.001	ref		<0.01
- Higher	3.4	(2.6–4.3)		2.65	(1.89–3.65)	
Hospital Unit						
- Not at risk	ref		0.15	ref		0.54
- At risk	0.8	(0.6–1.0)		0.91	(0.64–1.26)	
Daily washing of hands						
- ≤6 times a day	ref		0.95			
- ≥7 times a day	0.1	(0.8–1.3)				
Main reason for influenza vaccination adherence						
- To protect themselves/ To avoid mask wearing	ref		0.06	ref		0.54
- To protect patients	0.8	(0.6–1.01)		1.09	(0.81–1.48)	
Attitude to recommending influenza vaccination to patients						
- No	ref		<0.001	ref		<0.001
- Yes	6.35	(4.69–15.2)		3.45	(1.78–5.63)	
